# Considerations for Practice in Supporting Parental Bereavement in the Neonatal Intensive Care Unit—a Systematic Review

**DOI:** 10.1177/08258597231158328

**Published:** 2023-02-27

**Authors:** Jenna Lakhani, Cheryl Mack, Diane Kunyk, Janice Kung, Michael van Manen

**Affiliations:** 1 3158 University of Alberta, Stollery Children's Hospital, Edmonton, Canada; 2 3158 University of Alberta, Edmonton, Canada; 3 University of Alberta, Stollery Children's Hospital, John Dossetor Health Ethics Centre, Edmonton, Canada

**Keywords:** bereavement, neonatal intensive care, infant, death, parental

## Abstract

**Background:**

Parental bereavement after the death of an infant in a neonatal intensive care unit (NICU) is a complex and nuanced experience. Support from healthcare practitioners can have a significant impact on bereavement experiences in the short- and long-term. Although several studies exist exploring parental perceptions of their experience of loss and bereavement, there has not been a recent review of beneficial practices and common themes in the current literature.

**Objective:**

This review synthesizes empirical research to identify considerations that ought to guide the caregiving practices of healthcare professionals to support parental bereavement.

**Settings/subjects:**

Data was collected from studies identified in MEDLINE, Embase, and CINAHL. The search was limited to English-language studies describing parental bereavement in the NICU population from January 1990 to November 2021.

**Results:**

Of 583 studies initially identified, 47 studies of varying geographic locations were included in this review. Various themes surrounding healthcare support in parental bereavement were identified including ensuring the opportunity for parents to spend time caring for their child, understanding their perception of infant suffering, recognizing the impact of communication experiences with healthcare providers, and offering access to alternative means of support, all of which have been described as suboptimal. Parents generally want the opportunity to say goodbye to their infant in a private and safe space, be supported through their decision-making and be offered bereavement follow-up after loss.

**Conclusion:**

This review identifies methods of support in parental bereavement based on first-hand parental experiences and routine implementation of these strategies may be beneficial in supporting parents through their bereavement after the loss of a baby in the NICU.

## Introduction

Death is no stranger to the neonatal intensive care unit (NICU). Because of extreme prematurity, congenital anomalies, or other complex medical issues, some babies cannot survive despite medical interventions. Others are anticipated to have persistent health problems that severely impact their quality of life. Most NICU deaths result from withdrawing or withholding medical interventions.^1‐4^

End-of-life decision-making and subsequent neonatal death can create significant emotional turmoil for parents as they navigate their loss and associated bereavement.^5^ These parents need to live with the ethical decisions they make, recognizing that the decisions themselves have moral weight. The occasion of the birth of a child, which typically brings excitement and happiness, can instead be filled with grief, despair, and guilt.^6^ Recognizing the importance of, and supporting parents through, this loss is a challenging task for those working in the NICU. Healthcare practitioners need to have insights into parents’ experiences to ensure not only that they support decisions that forefront the interests of NICU infants, but also support the parents’ subsequent bereavement. It is important to understand what considerations ought to guide the caregiving practices of practitioners. Through consistent and compassionate bereavement care, practitioners will ultimately be able to enhance experiences for families as they navigate the complexities of infant loss.^5,7,8^

There have been no systematic reviews in the past decade exploring parental bereavement support in the NICU; however, there have been several related empirical qualitative studies. These studies provide understandings, reflections, and considerations regarding how healthcare practitioners can support families after a neonatal loss.

This review aimed to synthesize the findings of empirical NICU studies relating to parental bereavement and elaborate on considerations that healthcare practitioners can use to guide their caregiving practices and bereavement support.

## Methods

### Search Strategy

The search strategy for this review was developed with the assistance of a medical research librarian. A search of 3 electronic databases (MEDLINE, Embase, and CINAHL) was performed using a combination of keywords specific to the population (baby, neonate, newborn), the experience pertaining to loss (bereavement, death, dying, grief), and the type of care (palliative, terminal, end-of-life, comfort care). See Appendix 1 for details of the search terms and strategy used.

### Selection of Studies

This review included peer-reviewed articles published from January 1990 to November 2021. This date range was chosen to explore contemporary experiences surrounding parental bereavement in the NICU. After excluding duplicates and non-English studies, articles were eligible if based on the primary analysis of empirical data (such as interviews, surveys, and questionnaires). Systematic reviews, editorials, opinion pieces, conference abstracts, and articles published before 1990 were excluded. Grey literature was not reviewed; however, references from included studies were reviewed to ensure no key literature was missed.

**Figure 1.**
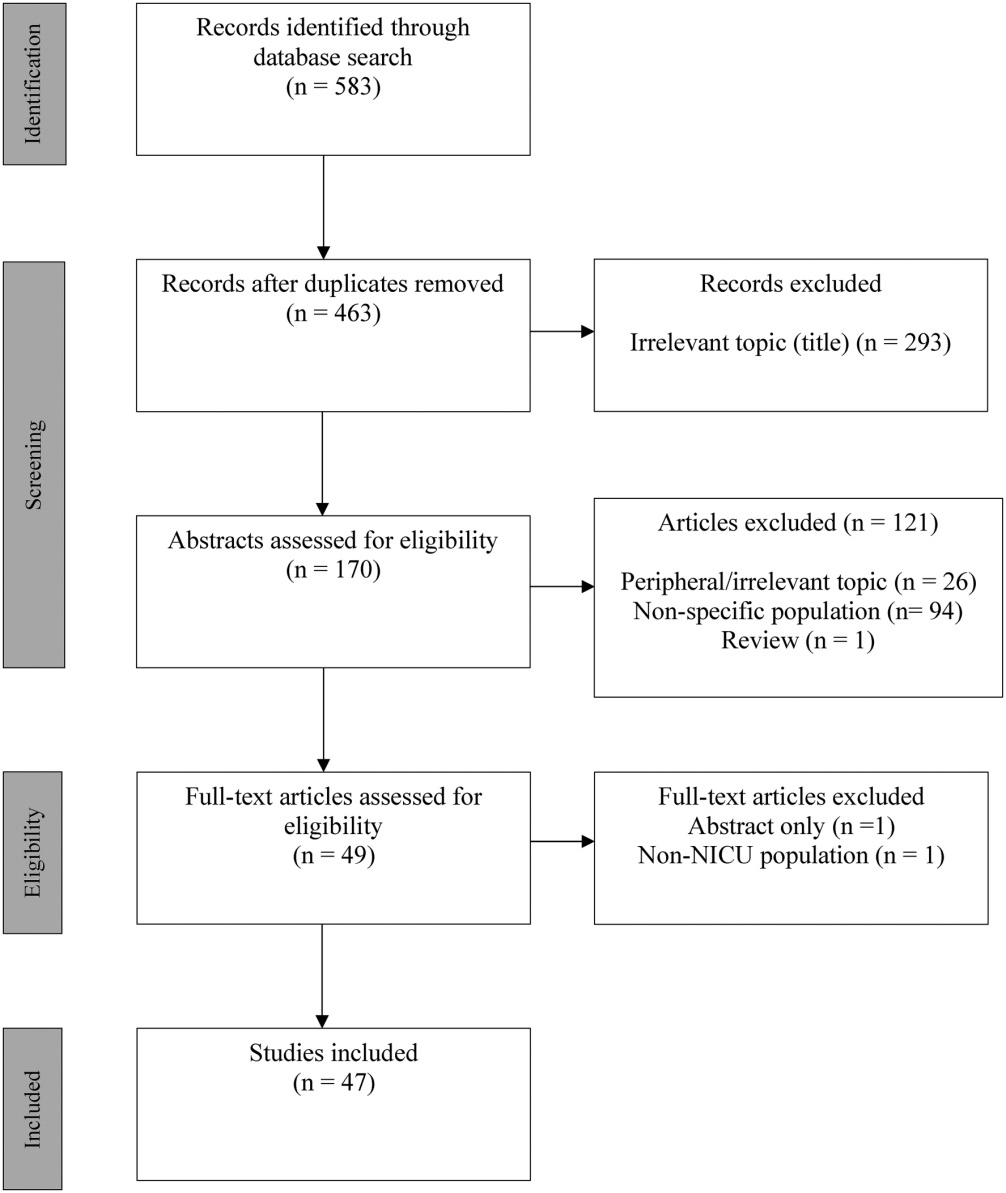
Article Selection Flow Diagram.

After abstract screening by 2 independent reviewers (JL/MvM), studies that involved general pediatric populations, combined pediatric and neonatal populations, or stillbirths and fetal death were excluded unless there was specific mention of independently analyzed NICU-related results. After screening full texts of the remaining results, 47 articles were included. Figure 1 illustrates the study selection process.

### Data Analysis

Each study was reviewed by 2 independent reviewers (JL/MvM). Descriptive data on the studies included first author, year of publication, location of study, journal of publication, research design (as described by the author), study purpose, and representative findings. The studies themselves were subjected to a qualitative description analysis, as described by Sandelowski, which seeks to summarize and present information in a coherent manner.^9^^,10^ For this study, the aim was to articulate themes that respond directly to the question: For parents experiencing the death of a child in a NICU, what considerations ought to guide the caregiving practices of healthcare professionals to support parental bereavement? In this way, the analysis was oriented specifically to the clinical practices of physicians, nurses, and others working in the NICU. The Mixed Methods Appraisal Tool (MMAT) was used to assess the quality of included studies.^1^^1^ An overall quality score was generated based on the percentage of quality criteria met, ranging from 0% to 100% such that the higher the percentage, the higher the study quality. Two reviewers (JL/MvM) independently assessed the quality of the studies and consensus was reached through discussion for any discrepancies.

## Results

A total of 583 studies were identified using the described search strategy. MEDLINE provided 282 studies, Embase provided 102 studies and CINAHL provided 197 studies.

Forty-seven studies (n = 47) were included in this review after assessing relevance. Geographic diversity was identified with the majority of studies being from the United States (N = 22) and others originating from the United Kingdom (N = 7), Australia (N = 5), Canada (N = 3), Jordan (N = 2), Netherlands (N = 2), Switzerland (N = 1), France (N = 1), Norway (N = 1), Ireland (N = 1), Israel (N = 1), and China (N = 1). Demographic information was variably presented in the reviewed papers and the majority did not specify details on participant demographics. However, 18 of the studies reported a participant population that largely included Caucasian mothers who were married at the time of infant death and with some level of higher education. Table 1 describes each study in detail, including the Mixed Methods Appraisal Tool (MMAT) score for each, which was calculated using the detailed MMAT assessment found in Table 2.

**Table 1.** Included Articles Describing Support of Parental Bereavement in the NICU.

Author (Year), Country	Design	Population (N)	Study purpose	Representative findings	MMAT score
Abdel Razeq & Al-Gamal^12^ (2018) *Jordan*	Phenomenology, thematic analysis: Interviews	12 parents	To understand bereavement and its associated meanings as lived and experienced by bereaved NICU mothers	- 3 main themes: (1) Longing and grieving, as natural emotional response to the loss; (2) Adaptive work of coping, as the mother internalized meanings to cope with the loss; and, (3) Moving forward but with a scar, as the mothers moved on with their lives while they carried the unforgettable memories of their newborn's death - Bereavement support services as essential services - Providers can support through understanding parents’ spiritual values and coping mechanisms	100%
Abdel Razeq & Al-Gamal^18^ (2021) *Jordan*	Phenomenology, thematic analysis: Interviews	12 parents	To understand the lived experiences of mothers surrounding the time of being informed of neonatal death in the NICU	- 3 main themes: (1) Minimize the hurt, which described how mothers intuited overprotection by their family; (2) The striking reality of death, which captured mothers’ distressing experiences while realizing the loss of their neonate; and, (3) Farwell my baby, which accentuated mothers’ needs and experiences while neonates’ bodies were honored and prepared for burial per the cultural norms in Jordan - Opportunities for parental involvement in care practices at the end-of-life - Need for specialized bereavement support at time of death	100%
Abraham & Hendriks^13^ (2017) *Switzerland*	Ethnography, content analysis: Interviews	20 parents	To illustrate the parental perspectives of those who lost an extremely premature infant in the hours/days after birth in the NICU, and how healthcare providers can facilitate bonding between parent and child during this time	- 2 main themes: (1) After the baby’ transfer to the NICU, a phase of uncertainty; and, (2) The end-of-life phase, when death is certain - Parental role may evolve from time of birth to time of death, from distant parenting to embodied parenting - Importance of parents being able to take on roles in providing care and parenting role - Ensuring privacy and opportunities to hold at end-of-life - Offering memory-making including photography is valued	100%
Akard et al^56^ (2018) *USA*	Qualitative description, content analysis: Focus groups	6 parents	To explore bereaved parents’ perceptions of legacy-making interventions after infant death	- 4 main themes: (1) Parents’ willingness to participate in legacy intervention; (2) Suggestions for feasible interventions; (3) Suggestions for acceptable interventions; and, (4) Parents’ perceived benefits of legacy-making - Parents support the use of legacy-making interventions, finding them to be feasible, acceptable, and beneficial - Bonding can continue after loss and can be shared with extended family members - Barriers include timing of approach, sensitivity to uncertain prognosis and logistical barriers	100%
Alexander^35^ (2001) *USA*	Qualitative case series, narrative: Interviews	4 parents	To understand the benefit of perinatal bereavement photography	- Bereavement photography should be included in loss policies and procedures - Photography should be personalized - Photographs can assist in grief - Sharing photographs with other children to help with sense-making and understanding	80%
Armentrout^47^ (2007) *USA*	Grounded theory: interviews	15 parents	To describe parents’ experiences about life support decisions, infant death, and lives thereafter To report basic social processes parents revealed as vital to sustaining infant's memory	- 3 main themes: (1) Facing the decision - no real choice, time with the baby; (2) Life goes on – listen to your heart, an abiding loss, not left out; (3) Lives forever changed – new perspective, preparing to meet again - End-of-life decision making complex, often require prognostic information from healthcare providers to contextualize the situation - Parents value choosing how to spend time with their infant after difficult decisions—seemingly where regrets were found in parental reflection - Concept of “shadow grief” - lessens with time but is never forgotten - Important for families to maintain infant's place as part of family long after death	100%
Armentrout^14^ (2009) *USA*	Grounded theory: Interviews	15 parents	To explore concepts identified by parents as factors in decision-making and on facilitators and barriers encountered in grieving process	- Varied experiences of end-of-life decision-making around in the NICU - Individualizing and personalizing care to each infant and family is appropriate - Parents may vary in their experiences of loss and bereavement - Cultural and social values may be expressed in coping strategies - Parents may appreciate encouragement to parent their child during NICU stay and end-of-life - Parental grief and loss become incorporated into parents ongoing lives and living with loss	100%
Armentrout & Cates^24^ (2011) ­*USA*	Qualitative description: Interviews	14 parents	To explore parental perceptions of experience of being informed of infant's inevitable death	- Lack of education in palliative care poses stress to NICU providers - Transparency of providers in decision-making highly valued - ABCDE approach: Advanced preparation, Building therapeutic environment, Communicating well, Dealing with patient and family reactions, and Encouraging and validating emotions - Simulations of difficult conversations may increase comfort levels of providers	20%
Baughcum et al^35^ (2017) *USA*	Qualitative content analysis: Interviews	45 parents	To examine parent perspectives of infant's end-of-life experience 3 months – 5 years after infant death	- Themes: (1) Parents as partners in care; (2) Communication with health-care team; (3) Relationship with staff; (4) Bereavement support - Bereavement resources highlighted by parents as influential - Highlight of importance in relationship of trust between parents and healthcare providers, staff members compassion, and parental involvement in infant bonding - Areas for improvement included importance of participation in care and having space to do so optimally, - Fathers often had higher satisfaction of care compared to mothers	100%
Baughcum et al^25^ (2020) *USA*	Mixed methods: Questionnaires, interviews	69 parents	To examine parent perceptions of infant end-of-life experiences (eg, symptom burden and suffering) and satisfaction of care in the NICU	- Perceptions of infant suffering relate to lower satisfaction with care in the NICU - Satisfaction with care relate to parents’ partnership in care, communication, relationships with staff, and bereavement support - Families have various needs that are variably met: keepsakes, acknowledgement of loss, ability to say goodbye, treating infant with dignity, involvement in infant care, availability of neonatologist, mitigation of suffering, contact with team after death (follow-up) - Providers should consider: palliative care basics, palliative care team, screening for mental health concerns, discuss coping strategies	100%
Blood & Cacciatore^29^ (2014) *USA*	Modified grounded theory, mixed methods: Questionnaires	181 parents	To examine the meaning, usefulness, and social context of bereavement photography in the eyes of NICU parents	- Photography can foster sense of ongoing connection and help other family members (eg, siblings) with understanding and coping - Photography practices should be individualized as some families and some cultures may not find them acceptable	60%
Bourque et al^48^ (2020) *Canada*	Mixed methods, convergent analysis: Field notes, questionnaires	8 parents 16 healthcare providers	To describe the ongoing involvement and bereaved parents’ perspectives in different activities in the NICU and providers who work with them	- Identified benefit of using resource parents (those who have previously experienced NICU hospitalization of infant) to improve care and experience for current parents - Themes identified: family perspectives, helping bereaved parents, improving system, giving back, promoting empowerment, targeting improvement in relationship with providers - Mutual beneficial relationship – gain control of experience, help with coping and healthcare providers get parental viewpoints directly	80%
Caeymaex et al^36^ (2013) *France*	Mixed methods: Interviews	78 parents	To investigate parents’ perceptions on type of involvement in end-of-life decision-making and effect on long-term grief	- Perceived suffering of infant associated with higher grief scores - Shared decision-making resulted in lower grief scores relative to medical or informed parental decision-making - Involvement in decision-making based on parental wishes	100%
Calhoun^19^ (1994) *USA*	Descriptive survey: Questionnaires	23 parents	To confirm appropriateness of nursing interventions that are commonly accepted as beneficial to parents during neonatal end-of-life	- Nursing interventions very impactful in parents’ experience at end-of-life - Parents valued interventions that dealt with acknowledgement of baby - Strategies employing educational/information/written material, general emotional support/communication, and general emotional support/interventions were valued - Parents may vary in how they grieve	60%
Clark et al^37^ (2021) *USA*	Qualitative cross-sectional survey: Questionnaires	67 parents	To explore associations between perceptions of infant suffering in NICU and parental adjustment after death	- Parents perceptions of infant symptoms and suffering were linked with levels of grief - Symptom management may support parental coping - Parents may vary in how they grieve	100%
Clarke & Booth^49^ (2011) *United Kingdom*	Descriptive survey: Questionnaires	13 parents 53 healthcare providers	To evaluate the opinions of bereaved parents on being provided a written summary of infant's care and death in NICU	- Most bereaved parents welcomed a copy of the detailed medical summary that provides a complete record of the clinical course, brief life, and terminal events in the NICU - Post-mortem and bereavement follow-up visits may be helpful for parents	60%
Cortezzo et al^30^ (2015) *USA*	Descriptive cross-sectional survey: Questionnaires	7 parents 104 healthcare providers	To determine perceptions of end-of-life care practices in NICU from neonatologists, practitioners, nurses, and parents and identify areas for improvement and involvement of palliative care team	- Most neonatologists and advanced practitioners comfortable with EOL care but think there would be benefit in having designated palliative care team - Parents stressed the importance of memory-making and follow-up/bereavement support - Palliative care team to spend time with the families enabling a true understanding of how significant the situation is and understanding their wishes/backgrounds would be beneficial - Value of consistency among providers, symptom management, early and clear communication, bereavement support, staff debriefing	60%
Cuisinier et al^51^ (1996) *The Netherlands*	Qualitative description comparison study: Questionnaires	142 parents	To investigate differences between parental grief following death of neonatal twin in comparison to singleton	- Bereaved twin parents did not differ in grief reactions from bereaved singleton parents in short or long them - Differences in grief experience as twin parents have to balance grieving loss of twin relative attachment to twin still living - Suggestions that parents make memories of twins separate and together - Important to document that live infant was product of twin pregnancy when dealing with parents and to examine potential consequences	60%
Currie et al^15^ (2019) *USA*	Qualitative description: Interviews	10 parents	To explore parents’ bereavement coping experiences after experiencing infant death in the NICU where palliative care was provided	- 2 main themes: (1) living with loss, as bereavement and grief over time, mental health challenges, spiritual suffering, personal growth after loss, and life changes after loss; and, (2) coping with grief over time as barriers to coping with loss and helpful strategies for coping with loss - Infant death was a tragic and life-changing loss for parents with feelings of loss evolving over time - Varied grief symptoms may be experienced - Parents navigate different parries to coping with varied supports	100%
Currie et al^26^ (2016) *USA*	Qualitative description, content analysis: Interviews	10 parents	To investigate how bereaved parents describe their experience related to NICU hospitalization, end-of-life care, and palliative care consultation	- Primary theme: life and death in the NICU environment with categories of ups and downs of parenting in the NICU, decision-making challenges in the NICU, and parent support - Parents appreciate the chance “to be a parent” regardless of how much they could be involved in the care of their infant, regardless off the strife endured, and regardless of how much time they ultimately had - Palliative care consultation often associated with some hesitancy but retrospectively appreciated as extra support - Standardized protocols involving palliative care consult may be of benefit	100%
Fortney et al^16^ (2020) *USA*	Qualitative description: Interviews, questionnaires	46 parents	To examine bereaved parents’ perceptions of infant suffering in the NICU	- 4 main themes: (1) the presence/absence of suffering; (2) indicators of suffering; (3) temporal components of suffering (trajectory); and, (4) influence of perceived suffering on parents, infants, and decision-making - Perceptions of suffering are informed by infant observations and information received from healthcare team members - Suffering perceptions influence parental decision-making	100%
Gilmour et al^39^ (2017) *Australia*	Retrospective cohort study: Chart review	46 infant charts	To characterise end-of-life care provided in a tertiary centre through assessing performance with known key components of palliative care	- Indicators of quality palliative care varied with some activities routine such as family meetings, social worker involvement, and memory-making opportunities while others were infrequent antenatal resuscitation planning, discussion of preferred location of death, and access to bereavement care - Respiratory symptoms, neurological symptoms, and pain were most commonly identified distressing symptoms - Neonatal staff have significant scope to improve end-of-life care by providing psychosocial, emotional, and spiritual supports	60%
Harrigan et al^20^ (1993) *USA*	Longitudinal study: Questionnaires	27 parents	To describe grief experiences of multi-gestation pregnancies after loss, to compare differences between mothers and fathers, to analyze the psychometric Perinatal Grief Scale for use in this population, and to develop foundation to study risk for pathological grief	- Grief scales as instruments reliable in population but sensitivity reduced - Coping strategy effectiveness dependent on time since infant death and length of infant's life - Longitudinal follow-up essential as grief is often prolonged when loss of 1 twin and surviving twin remains - Practitioners can help validate or refute relationships suggested in grief model and normalize development of perinatal grief	60%
Keim et al^21^ (2017) *USA*	Qualitative description, content analysis: Interviews	69 parents	To examine relationship between parent perceptions of infant suffering, parental distress, and the decision to have more children after infant death	- 4 main themes: (1) Impact of infant death; (2) Facilitators and barriers; (3) Timing and trajectories of decisions; and, (4) Not wanting to replace the deceased child - Mothers who had subsequent children following a loss had lower post-traumatic stress symptoms and fewer symptoms of prolonged grief - More positive NICU experiences were associated with willingness to have other children - Providers should not discourage parents from having other children and be cognisant of the advice they give	100%
Kochen et al^41^ (2021) *The Netherlands*	Qualitative exploratory study: Interviews	22 healthcare providers	To understand pre-loss care and challenges providers face in providing end-of-life care	- 3 main themes: (1) Sustaining hope versus realistic prospects; (2) Obtaining emotional closeness versus emotional distance; and, (3) Exploring emotions versus containing emotions - Providers weigh strategies based on their perceptions of parental needs, the situation, and their own competencies - Uncertainties as to whether the preloss care they provided constituted optimal care may exist - Working as a team is beneficial to learn from one another, prevent burnout and diversify care	80%
Kymre & Bondas.^31^ (2013) *Norway*	Phenomenology: Interviews	18 healthcare providers	To describe how nurses enact skin-to-skin care at the end-of-life	- Strong belief in the urgency of skin-to-skin care in providing mutual proximity and comfort for dying preterm newborns and their parents - Skin-to-skin care is seen as the preferred caring practice at end-of-life by nurses - Often more comfortable with less tubes/wires both for parents and for baby	100%
Leitao et al^44^ (2021) *Ireland*	Pilot workshop: Questionnaires	95 healthcare providers	To develop and implement a multidisciplinary perinatal bereavement care training to develop compassionate culture for bereaved parents	- TEARDROP program- Participants were satisfied with the workshop rating level of information and quality of teaching high	60%
Levick et al^32^ (2017) *USA*	Qualitative description: Questionnaires	36 families 11 healthcare providers	To describe an approach to delivering bereavement services to NICU families as well as education and support to NICU staff	- Bereaved parents and caregivers find meaning and purpose in the act of creating keepsake memories at the time of an infant's death - Individualized follow-up contacts by staff familiar with bereaved parents supports mutual healing - Families vary in their desired involvement in bereavement activities	60%
Lizotte et al^45^ (2020) *Canada*	Simulation session: Observation, scoring checklist	6 parents 13 healthcare providers 2 standardized actors	To identify core behaviors associated with good communication during and after unsuccessful resuscitation	- Participants judged as good communicators were more likely to introduce themselves, use the infant's name, acknowledge the parental presence, prepare the parents, stop resuscitation without asking parents, clearly mention death, provide or enable proximity, sit down, decrease guilt, permit silence, and have knowledge about procedures after death - Many simple behaviors, identified by parents and providers, are associated with good clinician-parent communication	80%
McHaffie^7^ (2001) *United Kingdom*	Qualitative description: Interviews	108 parents	To explore the perceptions of responsibility, burden and helpfulness or participation of parents in care withdrawal decisions	- Nurses play a key role in providing emotional support for families during the process of treatment withdrawal - Key elements include expressing compassion, demonstrating expertise and evidence, and consistency and honesty in information sharing - Parents valued nursing and staff attendance at funeral	100%
McHaffie et al^46^ (2001) *United Kingdom*	Qualitative description: Interviews	108 parents	To determine parental views on autopsy after treatment withdrawal	- Autopsy may be helpful even if cause of death known or expected as may provide additional information - Main reasons for declining autopsy were concerns about disfigurement, a wish to have the child left in peace, and a feeling that an autopsy was unnecessary - Parents did not appear to express regret about their autopsy decisions - Commonest cause for not receiving autopsy was not being asked - Consent and disclosure recommended to be done by neonatologist or trusted team member	100%
McHaffie et al^50^ (2001) *United Kingdom*	Qualitative description: Interviews	108 parents	To explore parents’ experiences of bereavement care after withdrawal of care in a NICU	- Appointments should be scheduled soon after death, certainly within 2 months of the death regardless of whether or not autopsy results are available; with the attending neonatologist; and, in a setting away from the hospital - Parents value efforts to find out how they are coping, honest information given sensitively; and, reassure where possible	100%
McHaffie et al^40^ (2001) *United Kingdom*	Qualitative description: Interviews	108 parents	To explore parents’ experiences with treatment withdrawal of their baby and the dying process	- Parents often had regrets on lacking information to support decision making and the length of time it took to make decisions to withdraw medical interventions - Length of the dying process could undermine parents confidence in their decisions or otherwise contribute to their distress - Medical predictions of time to death often were inaccurate - Better to give uncertain answer versus committing to a time frame of death after withdrawal	100%
Oreg^33^ (2020) *Israel*	Phenomenology: Testimonials, interviews	88 parents	To discover ritual structures and embodied processes that occur during milk donation after loss	- 2 main themes: (1) Extracting milk and continuing bonds with the lost baby; and, (2) The transformation in meaning through the milk extraction process - Bereaved mothers may experience ambiguity in loss as the combination of physical absence and psychology presence of their baby - Producing (through the process of lactating), extracting (through repeated pumping), and donating milk may be understood as a grief ritual, allowing mothers to maintain and reconstruct bonds with their babies - Milk seen symbolic gift from deceased infant and donating it to other babies could be the way of their own infant living on in others	100%
Pector^42^ (2004) *USA*	Mixed methods survey, grounded theory: Questionnaires	71 parents	To assess the experiences of bereaved parents of multiples with resuscitation and life-support discussions, the process of dying, and conversations with healthcare professionals about death	- Parents of multiples exhibit diverse and at times divergent opinions regarding resuscitation, coping styles, and approach decisions from varied perspectives - Careful, clear, and summaries of conversations supported parental decision-making - Language used to express empathy or support decision-making is meaningful - Time with the deceased was valued - Death notification is a sensitive task	40%
Rosenbaum et al^47^ (2015) *USA*	Randomized controlled trial, mixed effects model: Questionnaires	73 parents	To evaluate the impact of viewing bereavement support DVD on parental grief compared with standard bereavement care	- Limited numbers of parents watched the DVD - Analyses based on intention-to-treat did not demonstrate significant differences - Analyses of viewer compared to nonviewers showed differences in anxiety	40%
Rosenbaum et al^22^ (2011) *USA*	Case descriptions: Narratives	not specified	To increase awareness of spiritual and existential distress and to provide strategies to cope with end-of-life	- Families may rely on their faith and spirituality to assist them through end-of-life journey - Incorporating spiritual support can support parental bereavement - Spiritual support benefits from certified chaplain, clinician trained in spiritual care, or other individual with such expertise - Health care professionals may become more acquainted with the meanings of spiritual and religious concepts that emerge for patients and families in their clinical practice	20%
Shultz et al^38^ (2017) *USA*	Mixed methods: Questionnaires	67 parents	To compare reported/documented symptoms at end-of-life and associations with parent perceptions of infant suffering	- Parents report symptoms of respiratory distress, agitation, pain, and lethargy most commonly - Parental perceptions of infant suffering were correlated with total number of symptoms - Managing end-of-life symptoms is part of establishing “good death” - Discrepancies may exist between parents and medical team regarding perceptions of pain and other symptoms	60%
Skene^23^ (1999) *USA?*	Qualitative description: Interviews	9 parents	To evaluate existing research and contribution to current bereavement practices and illustrate individual nature of bereavement experiences by hearing individualized stories	- 10 main themes: (1) Relationship with the baby; (2) Staff; (3) Conflict over baby's treatment; (4) Information; (5) Discontinuation of treatment; (6) Time in NICU following the baby's death; (7) Holding and bathing the baby; (8) Support at home; (9) Partners; and, (10) Photographs and reminders - Individualization of care is needed for varied bereavement experiences	100%
Swanson et al^43^ (2009) *Australia*	Mixed methods study: Questionnaires, interviews	104 parents	To compare coping among couples who experience the death of a twin and who have a surviving twin or higher order multiple	- Gendered differences in reports of grief and depression - Parental spiritual beliefs may increase after loss, with some parents turning to spiritual support - Parents value support from partners, talking (especially with each other), family and friends offering acknowledgement/understanding, spiritual and religious beliefs, surviving twin and other children	80%
Swanson et al^52^ (2002) *Australia*	Mixed methods study: Questionnaires, interviews, focus groups	66 parents	To explore the nature of bereavement in losing one or more infants in a multiple pregnancy, how mothers cope and how they can be better supported	- Mothers show higher levels of active grief than difficulty coping, and more difficulty coping than despair - Spiritual beliefs may increase after loss, with some parents turning to spiritual support - Helpful themes: surviving children, acknowledgement from family and friends, support from father of twins, spiritual/religious beliefs - Unhelpful themes: Unacknowledged grief, insensitive comments, social workers’ interaction and attempt to give advice on how to grieve, seeing live twin pairs, lack of support from twin father, being blamed	80%
Thornton et al^27^ (2021) *Australia*	Grounded theory: Interviews	18 parents	To explore the significance and impact of memory-making on parents’ experience at end-of-life	- Core category of “Affirmed parenthood” was underpinned by: (1) Creating evidence; (2) Needing guidance; and, (3) Being a parent - Parents value being supported to have contact, engage with, and provide care for their baby in end-of-life care as critical elements of memory-making and being a parent in a situation where many “normal” parenting activities were not possible	100%
Thornton et al^34^ (2020) *Australia*	Grounded theory: Interviews	18 parents	To explore the significance and impact of memory-making on parents’ experience of loss following neonatal loss	- "Creating evidence” was a key theme of memory-making involving taking photographs, creating mementos, as well as involving friends and family during their baby's time in the NICU - Memory-making may affirm the life of the baby and the role of the parents to ultimately support parental bereavement - Creating evidence does not just take the form of tangible artefacts but also social interactions and other memories	100%
Welborn^53^ (2012) *USA*	Phenomenology: Interviews	21 parents	To explore the lived experience of milk donation in bereaved mothers	- 4 main themes: (1) Identifying as a mother, grieving the loss of motherhood; (2) Meanings associated with the experience of pumping milk; (3) Finding meaning in and integrating the experience of perinatal loss; and, (4) The importance of addressing lactation with bereaved mothers - Bereaved mothers benefit from support and education regarding lactation following loss - Pumping milk was a way of grieving loss, donating milk was a way of accepting and healing from loss - Advocacy for more holistic approach to lactation including physiological and emotional aspects, and supporting bereaved mothers in lactating needs	100%
Williams et al^17^ (2009) *Canada*	Qualitative description: Interviews, questionnaires	11 parents	To develop and pretest a questionnaire on withdrawing life-sustaining interventions in NICU based on bereaved parents’ experiences	- 6 domains: (1) Communication; (2) Quality of care; (3) Quality of life; (4) Shared decision-making; (5) Withdrawal of life-sustaining treatment process; and, (6) Bereavement care - Withdrawal of life-sustaining treatment process needs were identified as most likely met while those related to quality of care and bereavement care were not consistently met	100%
Wool et al^8^ (2018) *USA*	Descriptive survey: Questionnaires	405 parents	To assess parental satisfaction with care received in the context of a life-limiting fetal diagnosis and subsequent birth	- Parents value being supported by their healthcare team and describe components of care - Predictors of parent satisfaction include: provision of compassionate care, physicians taking time to talk to parents, and help with emotional coping - Parents value clinicians listening to them, incorporating their perspectives in decision-making, and supporting choices (continuing pregnancy with life-limiting condition) - Bereavement support available to help parents cope which is highly valued – includes navigating anticipatory grief after infant is born	60%
Yam et al^28^ (2001) *China*	Qualitative description, content analysis: Interviews	10 healthcare providers	To explore experiences of neonatal nurses caring for dying infants, their perceptions on palliative care and factors influencing care	- 8 categories: (1) Disbelieving; (2) Ambivalence and helplessness; (3) Protecting emotional self; (4) Providing optimal physical care to infant; (5) Providing emotional support to family; (6) Expressing empathy; (7) Lack of knowledge and counseling skills; and, (8) Conflicting values in care - Need for professional development in palliative care nursing education - Implementation of bereavement support team may support practice improvements - Small changes can make a big difference: flexible visiting hours, multipurpose rooms for family members, private room for family privacy and religious activities	100%

Abbreviations: NICU, neonatal intensive care unit; TEARDROP, Teaching, Excellent, pArent, peRinatal, Death-related inteRactions, tO, Professionals.

Seven thematic considerations with relevant subthemes were derived from the analysis of included studies (see Table 2).

**Table 2.** Mixed Methods Appraisal Tool (MMAT).

	Paper	1.1	1.2	1.3	1.4	1.5	Total Score
1	Abdel Razeq et al 2018	Y	Y	Y	Y	Y	100%
2	Abdel Razeq et al 2021	Y	Y	Y	Y	Y	100%
3	Abraham et al 2017	Y	Y	Y	Y	Y	100%
4	Akard et al 2018	Y	Y	Y	Y	Y	100%
5	Alexander 2001	Y	-	Y	Y	Y	80%
6	Armentrout et al 2007	Y	Y	Y	Y	Y	100%
7	Armentrout et al 2009	Y	Y	Y	Y	Y	100%
8	Armentrout et al 2011	-	-	Y	-	-	20%
9	Baughcum et al 2017	Y	Y	Y	Y	Y	100%
10	Baughcum et al 2020	Y	Y	Y	Y	Y	100%
11	Blood et al 2014	Y	-	Y	-	Y	60%
12	Bourque et al 2020	Y	Y	Y	Y	-	80%
13	Caeymaex et al 2013	Y	Y	Y	Y	Y	100%
14	Calhoun 1994	Y	-	Y	-	Y	60%
15	Clark et al 2021	Y	Y	Y	Y	Y	100%
16	Clarke et al 2011	Y	-	Y	-	Y	60%
17	Cortezzo et al 2015	Y	-	Y	-	Y	60%
18	Cuisinier et al 1996	Y	Y	Y	-	-	60%
19	Currie et al 2019	Y	Y	Y	Y	Y	100%
20	Currie et al 2016	Y	Y	Y	Y	Y	100%
21	Fortney et al 2020	Y	Y	Y	Y	Y	100%
22	Gilmour et al 2017	Y	Y	Y	-	-	60%
23	Harrigan et al 1993	Y	-	Y	-	Y	60%
24	Keim et al 2017	Y	Y	Y	Y	Y	100%
25	Kochen et al 2021	Y	-	Y	Y	Y	80%
26	Kymre et al 2013	Y	Y	Y	Y	Y	100%
27	Leitao et al 2021	Y	-	Y	-	Y	60%
28	Levick et al 2017	Y	-	Y	-	Y	60%
29	Lizotte et al 2020	Y	Y	Y	Y	-	80%
30	McHaffie 2001	Y	Y	Y	Y	Y	100%
31	McHaffie et al 2001	Y	Y	Y	Y	Y	100%
32	McHaffie et al 2001	Y	Y	Y	Y	Y	100%
33	McHaffie et al 2001	Y	Y	Y	Y	Y	100%
34	Oreg 2020	Y	Y	Y	Y	Y	100%
35	Pector 2004	Y	-	-	-	Y	40%
36	Rosenbaum et al 2015	Y	Y	-	N	N	40%
37	Rosenbaum et al 2011	-	-	Y	-	-	20%
38	Shultz et al 2017	-	-	Y	Y	Y	60%
39	Skene 1999	Y	Y	Y	Y	Y	100%
40	Swanson et al 2009	Y	-	Y	Y	Y	80%
41	Swanson et al 2002	Y	Y	Y	Y	-	80%
42	Thornton et al 2021	Y	Y	Y	Y	Y	100%
43	Thornton et al 2020	Y	Y	Y	Y	Y	100%
44	Welborn 2012	Y	Y	Y	Y	Y	100%
45	Williams et al 2009	Y	Y	Y	Y	Y	100%
46	Wool et al 2018	Y	-	Y	-	Y	60%
47	Yam et al 2001	Y	Y	Y	Y	Y	100%

1.1. Is the qualitative approach appropriate to answer the research question?

1.2. Are the qualitative data collection methods adequate to address the research question?

1.3. Are the findings adequately derived from the data?

1.4. Is the interpretation of results sufficiently substantiated by data?

1.5. Is there coherence between qualitative data sources, collection, analysis and interpretation?

Abbreviations: Y, yes; -no; MMAT, Mixed Methods Appraisal Tool.

**Table 3.** Primary and secondary themes.

*Parents may experience a manifold of different emotions anticipating and following the death of their child* 1. Grief as an emotion and as a constellation of emotions 2. Evolution of grief and bereavement 3. Individualizing bereavement care
*Parents may live with a cascade of different losses, including the loss of their child, shaping their bereavement* 4. Complexity of loss and losses 5. Parental presence and involvement in care 6. Meaning and memory-making
*Parents live with their perspective of the care their child and they received through their* neonatal intensive care unit (*NICU) journey* 7. Perceptions of pain and suffering 8. Supportive communication 9. Care at the end-of-life
*Parents may benefit from a variety of different supports in anticipation of bereavement* 10. Spiritual/religious support 11. Pediatric Palliative care and bereavement services 12. NICU team support
*Parents live with their decisions in bereavement; with time, they may question, re-evaluate, or come-to-terms with the decisions they made* *decision-making* 13. Individualizing involvement in shared decision-making 14. Autopsy
*Parental bereavement benefits from support beyond the NICU* 15. Physical and mental health outcomes 16. Bereavement follow-up 17. Future pregnancies/children
*Understandings are needed for unique NICU bereavement experiences* 18. Loss of single twin 19. Breastmilk production after loss

### Parents may Experience a Manifold of Different Emotions Anticipating and Following the Death of Their Child

Shared between the included studies is the consideration that parents vary in how they identify and resolve their emotional reactions to the loss of their baby. Grief was described as a predominant constellation of emotions experienced by parents in coping with the acute loss of their baby. From a psychological perspective, grief may be specified as those emotions associated with loss (compared to mourning as those actions resulting from grief).^12^ Encircling grief is uncertainty, disappointment, shame, guilt, suffering, and helplessness.^13‐17^ During an infant's end-of-life, parents often need to balance attachment to their infant with the impending separation.^17^ Following, grief may be described as a social phenomenon that is resolved through interactions with various support systems.^18^ Severity of grief can be linked to the baby being acknowledged as a separate entity and the duration of postnatal bonding time.^19‐21^ Finally, grief may be conceptualized as evolving through stages: denial, anger, bargaining, depression, and acceptance.^22^ With a neonatal loss, parents may experience these stages partially or fully, in a variety of sequences, and acceptance may never fully transpire.

Bereavement, like grief, is highly individual, yet also a recognizable human experience, as parents live with the loss of their child over time.^12‐15^ Living with loss is a sense-making experience as parents come to terms with what their child's life was like their presence for their child as parents, and other value-judgments.^15^ Healthcare professionals may anticipate, recognize, and respond to parental grief to support evolving and delayed emotional reactions that accompany bereavement.^12,14^ Focusing on attaching positive meaning to the life lost and maintaining hope—that suffering has ended or that the infant's life had purpose—can support parental bereavement.^12,14,22^ However, this consideration cannot be generalized because parents’ wishes, values, and other moral beliefs may vary.^13,14^ Generalizations can lead to a disconnect between healthcare professionals and parents by influencing opinions on what the perceived right action may be in a difficult scenario.^23^

### Parents may Experience a Cascade of Different Losses Shaping Their Bereavement and Healthcare Professionals can Affect This Burden of Loss

The reviewed studies reflect the broader NICU literature describing the complexity of losses parents may experience when their child requires hospitalization in a NICU: physical separation in the first days of life, parental role interruptions, and so forth.^14,24‐27^ Parental bereavement unfolds against the backdrop of parents’ NICU experiences, which can include loss experiences before, and in addition to, the death of their child.^13,14,26^ Activities of healthcare professionals that support parental involvement may potentially alleviate some of what is lost in these situations.^13,14,26‐28^ From the void of loss, healthcare professionals can support meaningful moments and memory-making, which is highly valued as part of bereavement.^13‐15,23,27,29,30‐35^ Parents seemingly appreciate any opportunity to be involved in their infant's care including bathing, dressing, diapering, and taking on other parental responsibilities.^13,14,26,27,35^ Photographs and other media provide parents with tangible objects to relieve their worry about their baby's memory fading over time.^36^ It also allows them to share memories and experiences with others who may not have been present for this difficult journey.^13,29,34^ Other objects include hand/foot molds, clothing, identification bands, or blankets.^34^ Photographs taken after death can depict loss and provide meaning to the experience as one of the most common forms of memory-making at neonatal end-of-life.^36^ Individualizing the extent of bereavement care is essential as some parents may not be comfortable with these opportunities and can feel pressure if nurses elude to any associated regrets that may arise in the future.^23,32,34,35^

### Parents Live With Their Perspectives of the Suffering Their Child Experienced and Their Communication Experiences With Healthcare Professionals

Families carry with them what happened in the NICU.^14,26^ In several studies, associations between the perception of infant suffering and the degree of parental grief, adjustment difficulty, and posttraumatic stress symptoms were clearly illustrated.^16,25,37‐39^ Parents perceive suffering through their infants’ symptoms and based on information received from healthcare providers.^16,25,26^ Distressing symptoms include skin breakdown, respiratory distress, pain, agitation, lethargy, feeding difficulties, and edema.^16,26,39‐41^ A common finding illustrated that parents more often perceived suffering in their baby compared to healthcare professionals.^30,39^

How healthcare providers communicate with parents is highly consequential: the language used needs to be sensitive yet appropriate to the situation.^13,15,24,41,42^ Statements that presume understanding such as “I know how you feel” can be distressing. ^15,24,42,43^ Though parents often want to be told medical information in a sensitive, yet straightforward manner, ambiguity may be the reality regarding the timing of death and other less predictable events.^41‐43^ A lingering prolonged death may undermine parents’ confidence in their decisions.^41^ Multiple studies portray the difficulties that arise for parents at the last moments of a baby's life in the NICU. Ensuring privacy, comfort, and security are generally valued.^13,18,28,31,36^ Healthcare professionals can support families by being deliberate yet flexible in end-of-life care practices.^15,36,43^ Some parents may not be comfortable being present for the final moments of their child's life. In these cases, parents may still find comfort in speaking to their baby before death, holding their baby while still warm, or entrusting the care of their child to a friend or healthcare provider.^27,36,43^ For others, holding their child is deeply meaningful.^14,27,31^ Removing monitoring and medical devices may support a family to focus on their baby.^14,27,31^ Managing end-of-life symptoms can help parents achieve a “good death” for their child.^39^ Providers should be aware that the suffering perceived by parents can contribute not only to their end-of-life decision-making, but also to how they ultimately live with their decisions in bereavement.^16^

### Parents may Benefit From a Variety of Different Supports in the NICU in Anticipation of Bereavement

Several studies explored the support parents value when navigating the emotional complexities of neonatal end-of-life. The benefits of spiritual care, including prayer, rituals, clergy, and belief in the transcendent quality of the parent-infant relationship are well established; however, healthcare professionals may feel uncomfortable broaching such subjects. ^12,14,22,32‐34,44^ Education in spiritual and religious concepts can help diminish the disconnect between parental values and provider discomfort.^22^ In comparison, palliative care consultation can offer expertise in psychosocial support, symptom management, and coordination of care.^25^ Neonatal practitioners find benefit in palliative care team members’ ability to learn about each family's individualized priorities, circumstances, and wishes.^30^ Palliative care involvement is associated with an increase in the number of family meetings, the identification of more symptoms, and creating symptom management plans.^40^

Developing bereavement expertise among a subset of NICU team members can support the provision of palliative care.^32,42^ Parents often look to nurses for support as the time spent at the bedside helps build trust and effective communication.^7,14,19,26,35^ Tailored education with a focus on communication, coping strategies, and enhanced exposure may help providers develop skills in enabling quality of care and supporting grief, navigating anxiety, and overcoming inexperience in end-of-life scenarios.^12,13,24,28,42,45,46^ Workshop initiatives have been piloted including the Teaching, Excellent, pArent, peRinatal, Death-related inteRactions, tO, Professionals (TEARDROP) program, which uses a Structured, Clinical, Objective, Referenced, Problem-oriented, Integrated and Organized (SCORPIO) approach to teach providers bereavement care strategies.^45^

### Parents Live with Their Decisions in Bereavement — with Time, They may Question, re-Evaluate, or come to Terms with the Decisions They Made

In bereavement, parents live with their decisions from the NICU.^14,26^ As such, it is important to consider how parents are supported in their decision-making. Studies describe various models of decision-making including shared decision-making (parents and providers approach a decision together), medical/paternalistic decision-making (a decision made exclusively by healthcare providers), and informed parental/autonomous decision-making (parents make a decision after a provider explains the medical information).^37,43^ Shared decision-making is generally recognized as the most appropriate approach as it benefits from healthcare providers’ medical expertise and family members’ values and beliefs.^37^ These conversations provide opportunities to clarify understanding, align clinical care with care goals, and express compassion to families.^7,8,43,47^ Parents should have the opportunity to express their preferences for their role in decision-making.^37^ Continuity of care and relationships, veracity in communication, expressions of empathy, expertise, and use of evidence, and clear documentation have all been identified as beneficial.^7,8,24‐26,39,43,46^ Parents who experience shared decision-making seem to have less grief than those who experience medical or parental decision-making.^37^

Included studies also highlighted how parents not only live with their decisions regarding goals of care and medical interventions, but also those decisions impacting how present they were for their child engaging in parenting actions, memory-making, and involving others in the lives of their child.^14,15,26,27,34,47^ There are also those decisions specific to end-of-life that parents live within their bereavement. For example, whether to pursue an autopsy can be a challenging decision. Often the infant's cause of death is known; however, errors in diagnoses may be found in postmortem evaluations.^48^ Parents may find benefit in an autopsy's ability to assess obstetrical and genetic risks for future pregnancies.^48^ Information may also be found that validates end-of-life decisions. These benefits are not always encountered as providers may be hesitant to offer an autopsy. ^48^

### Parental Bereavement may Benefit From Support Beyond the NICU

It is well recognized that parents whose children died in the NICU have increased risks for physical health concerns, including hospitalizations and higher mortality.^15,18^ Bereaved parents also experience higher rates of clinical depression, anxiety, and post-traumatic stress.^38,49^ Screening for pre-existing mental health concerns and developing support strategies before an anticipated death can be beneficial.^25^

To support mental health, professional counselling, support groups, peer counselling, and community physicians have all been described as potentially helpful to support acceptance and adaptation to loss.^15,33,40,50^ For some families, spiritual or religious supports are significant; however, finding adequate support can be challenging.^14,15,27,49^ Additionally, there is a timeliness to the extent, interval, and duration of support.^51^ Parents may experience an abrupt loss of support following the death of their child as they lose the support of the NICU.^30^ Initiation of timely supportive care to where parents are in their grieving beyond the NICU is needed.^17,30^

Most parents will attend a follow-up appointment with the healthcare team if offered.^52^ It would seem that these follow-ups should be scheduled 6 to 8 weeks after the death and in a location outside of the NICU.^52^ These follow-up visits can help provide answers to parents’ questions and reassurance about end-of-life decision-making.^52^ Other options for bereavement follow-up involve familiar staff individualizing follow-up contact over time, sending a card on the first anniversary of the infant's death, and telephone calls by the involved neonatologist.^32^ Parents described the benefit from being copied on the written summary from the NICU.^51^ Neonatologists should ensure that these summaries maintain sensitivity, use the baby's chosen name if available, and simplify terms to ease parental understanding.^51^ Deficiencies in bereavement support include medical follow-up, autopsy discussion, sibling grief management, marital concerns, and expectations surrounding return to normalcy.^48,49^

### Understandings are Needed for Unique NICU Bereavement Experiences

Bereavement following the loss of 1 infant from a multiple gestation pregnancy is a unique phenomenon that may be encountered in the NICU. Losses may occur before or following birth leading to varied bereavement experiences.^14,44,53,54^ At times, NICU healthcare providers may fail to acknowledge pregnancy loss as the focus is diverted to the surviving infant receiving care.^14^ It is essential to document and communicate pregnancy loss to NICU team members to acknowledge this grief potential.^53^ It can be challenging for parents to balance grieving the deceased while maintaining attachment to the living.^53^ Grief may be compounded by a failure to acknowledge the loss and/or by associated challenges of the surviving sibling.^14,20,54^ Some families will request aggressive treatment for the surviving child despite poor prognosis, while others are willing to stop when treatment is considered futile.^43^ The complexities of loss and survival of siblings may be challenging for families to navigate, recognizing mothers and fathers may vary in their bereavement.^44^

Another unique phenomenon surrounding neonatal loss involves lactation. Studies reflect a movement to advocate for a holistic approach to supporting mothers with lactation through their bereavement.^55^ Pumping milk has been an outlet for grief and milk donations have been associated with acceptance and healing.^42,53^ Mothers may experience several emotions when pumping after neonatal death including sadness, emptiness, anxiety, and relief.^53^ They may find strength in believing that donating milk will help sustain their child's legacy, maintain their parental identity, and keep the memory of their infant alive through helping others.^33,55^

Bereavement experiences can continue into future pregnancies.^12^ The decision to have another child after a neonatal loss may be very difficult.^21,49^ However, it would seem most parents have additional children following their loss and this is associated with fewer symptoms of prolonged grief, and posttraumatic stress.^12,21^ It is suggested that healthcare providers should not discourage or provide recommendations for the timing of future pregnancies.^21^ Instead, they should approach such discussions nonjudgmentally with compassion and sensitivity.

## Discussion

### Key Concepts of Supporting Parental Bereavement in the NICU

Through conducting this review, several themes exploring parental bereavement after the death of an infant in the NICU were identified based on primary data gathered from direct communication with bereaved parents. Although the burden of neonatal death and the impact of provider support is well-established in the literature, this review revealed that the ability of parents to spend time caring for their child, their perception of infant suffering, their communication experiences with healthcare providers, and the access to alternative means of support is often suboptimal. These themes can be a starting point in enhancing the support healthcare providers deliver to parents experiencing the loss of an infant and their subsequent bereavement. Based on the reviewed studies, implementing additional methods of support including access to spiritual and palliative care, shared decision-making, lactation support, and ensuring ongoing bereavement support may have significant benefits. Although general themes have been identified, it is important to appreciate that bereavement care both during and after an infant's death should be individualized. Different values, cultural backgrounds and family circumstances can contribute to different wishes and coping mechanisms for a family during their bereavement. Recognizing these nuances, healthcare providers need to customize their approaches to ensure optimal outcomes.

### Gaps in the Literature

There is an abundance of literature surrounding bereavement care after pregnancy loss, stillbirth, and perinatal death, including the death of a fetus between the 22nd full week gestation (or 500 g estimated weight) and 7 days after birth.^57^ While some of this literature appeared to include parental perspectives of those whose child died in the NICU, issues unique to perinatal bereavement for such families were left unarticulated. There is a clear need for literature that focuses exclusively, or at least forefronts some of the perspectives of parents whose child received medical interventions and admission to a NICU. The notion of perinatal death includes experiences that may resemble yet also differ from those occurring in the NICU.

Given that the studies reviewed reported specific demographic information on participants being Caucasian, educated, English-speaking and married, uncertainty remains in the influence of familial and cultural diversity in contributing to parental bereavement after a neonatal loss. Broader populations including those who are geographically distanced, recent immigrants, or less educated may have different values in bereavement practices, satisfaction, access to care and involvement in decision-making. An attempt to close this research gap by diversifying inclusion in related research studies could impactfully contribute to the current literature.

### Unresolved Tensions in the Literature

Although several conclusions can be formed through similarities found in the reviewed articles, some discrepancies remain. Cultural differences when providing bereavement care are a complex area to navigate. In some cultures, burial and body preparation as well as seeing the body after death is not considered appropriate; however, mothers often find it difficult not to have this opportunity and the literature strongly supports inviting parents to participate.^12^ Additionally, in similar cultures, the delivery of news about prognosis and goals of care are often relayed through family members and not the mother directly—which is contradictory to what is considered beneficial to parents as described in most papers.^12^ Mothers often find lactation difficult when their neonate is critically ill and despite several papers focusing on pumping and donating milk as an emotional release, some mothers find the pressure to pump and produce milk adds to their burden of stress.^13^ Despite education surrounding communication in goals of care and end-of-life being described as rare and suboptimal, 1 paper explores how practitioners feel confident in end-of-life care.^15^ Majority of other papers describe practitioners’ desire for further education and training in this area of neonatology.^16,30^ Moreover, despite the abundance of literature describing the benefit of pediatric palliative care consultations in the NICU, 1 paper illustrated palliative care as being nonsignificant when reporting parental satisfaction.^40^ These specific controversies remain unresolved but are crucial to consider when establishing bereavement practices and further emphasize the importance of individualizing care based on each family's beliefs and values.

### Limitations of Research

The majority of included studies were small-scale studies identifying themes and conclusions based on local trends. A narrative approach was chosen for this review to capture the diversity of research methods employed. The analysis also included the use of the MMAT demonstrating the findings of the study as credible, meaningful, and relevant to neonatal practice. Given the small-scale studies and results from predominantly developed English-speaking countries, caution should be exercised regarding generalizations. However, appreciating that several studies identified similar conclusions, despite the results of each being subjective and individualized, the commonality of these opinions strengthens the review's findings.

### Opportunities for Future Research, Medical Education, and Practice Change

Future research into parental bereavement for those who have lost an infant in the NICU should include exploring the contribution of culture on optimal bereavement support. The geographic diversity of studies in this review alludes to the individualization of care for each family, but also for each culture—and investigating these nuances may be beneficial in further tailoring neonatal bereavement care.

Other areas for research involve expanding on education initiatives in equipping healthcare practitioners with communication and support skills to assist families in their bereavement as well as ensure they are getting the support they need, especially if there is a history of pre-existing mental health concerns. Additionally, understanding the influence of autopsy on grief, closure, and future pregnancies could indicate the importance of offering postmortem examination in both confirming diagnoses as well as providing closure. Obtaining longitudinal follow-up data would also be a useful contribution to the literature as understanding how bereavement experiences impact families long-term can help prioritize areas for improvement.

## Conclusion

This review is the first of its kind in the past decade to explore current perspectives on parental bereavement and what considerations ought to guide the caregiving practices of healthcare professionals. Various methods of support have been identified based on first-hand parental experiences and routine implementation of these strategies may be beneficial in supporting parental bereavement. Future studies exploring the success of implementing these recommendations from parents’ experiences can help determine the usefulness of these strategies and provide the next steps to further enhance parental bereavement support in the NICU.

## Appendix 1: Search Strategy Outline

1. **MEDLINE/OVID:**

a. Exp infant, premature/ OR exp infant, newborn/ OR neonat* OR perinat* OR baby OR exp premature birth/ OR babies OR exp infant, diseases/ OR newborn* OR exp intensive care, neonatal/ OR NICU or exp intensive care units, neonatal AND

b. (Bereave* OR grief OR grieving OR loss) adj3 (Parent* OR caregiver* OR mother* OR father*) AND

c. Exp palliative care/ OR palliative OR exp palliative medicine/ OR comfort care OR exp patient comfort/ OR terminal* OR exp terminal care/ OR “end of life” OR exp death/

*1990-current, English language, excluding comments, editorials, reviews or systematic reviews

= 282 results

1. 2. **EMBASE:**

a. Exp neonatal intensive care unit/ OR exp prematurity/ OR neonat* OR perinat* OR exp newborn/ OR baby or exp baby/ OR babies OR exp newborn period/ OR newborn OR exp newborn death/ OR exp newborn/ OR exp newborn mortality/ OR newborn intensive care or NICU AND

b. (Bereave* OR grief OR grieving OR loss) adj3 (Parent* OR caregiver* OR mother* OR father*) AND

c. Palliative OR exp terminal care/ OR exp patient comfort/ OR comfort care OR terminal* OR “end of life”

*1990-current, English language, excluding editorials, reviews or systematic reviews

*= 102 results*

3. **CINAHL**

a. (MH “Intensive Care Units, Neonatal”) OR (MH “Neonatal Intensive Care Nursing”) OR (MH “Intensive Care, Neonatal+”) OR (MH “Infant, Newborn, Diseases+”) OR (MH “Perinatal Death”) OR (MH “Infant, Newborn+”) OR (MH “Infant, High Risk”) OR (MH “Infant Mortality”) OR “neonat*” OR “perinat*” OR (MH “Infant, Newborn, Diseases+”) OR “baby” OR (MH “Infant, Premature”) OR (MH “Infant, Postmature”) OR (MH “Infant, Premature, Diseases+”) OR (MH “Infant Death+”) OR “babies” OR “newborn*” OR “NICU” AND

b. (Bereave* OR grief OR grieving OR loss) N3 (Parent* OR caregiver* OR mother* OR father*) AND

c. (MH “Palliative Medicine”) OR (MH “Palliative Care”) OR (MH “Terminal Care+”) OR “palliative” OR (MH “Comfort Care (Saba CCC)”) OR “comfort care” OR “terminal*” OR “"end of life"”

*English language, peer reviewed journal, 1990-present, abstract available

*= 197 results*
